# Resveratrol and lycium barbarum polysaccharide improve Qinling giant panda (*Ailuropoda melanoleuca Qinlingensis*) sperm quality during cryopreservation

**DOI:** 10.1186/s12917-021-03122-2

**Published:** 2022-01-07

**Authors:** Ruixue Zhang, Hemeng Dong, Pengpeng Zhao, Chunmei Shang, Hang Qi, Yongjie Ma, Chuxi Gao, Danhui Zhang, Jiena Shen, Yinghu Lei, Yaping Jin, Pengfei Lin

**Affiliations:** 1 College of Veterinary Medicine, Northwest A & F University, Yangling, 712100 Shaanxi China; 2 Key Laboratory of Animal Biotechnology, Ministry of Agriculture and Rural Affairs, Northwest A&F University, Yangling, 712100 Shaanxi China; 3 Research Center for The Qinling Giant Panda, Rescue Base of Rare Wild Animals in Shaanxi Province, Louguantai, 710402 China

**Keywords:** Qinling giant panda, cryopreservation, sperm quality, antioxidants, freezing medium

## Abstract

**Background:**

Semen cryopreservation has become an essential tool for conservation efforts of the giant panda (*Ailuropoda melanoleuca*); however, it is severely detrimental to sperm quality. Evidence has shown that antioxidants have the potential to reverse cryopreservation-induced damage in sperm. The purpose of this study was to screen effective antioxidants that could retain sperm quality during cryopreservation and to determine the optimal dose. Seven antioxidant groups, including resveratrol (RSV = 50 μM, RSV = 100 μM, RSV = 150 μM), lycium barbarum polysaccharide (LBP = 2 mg/mL, LBP = 4 mg/mL), laminaria japonica polysaccharides (LJP = 1 mg/mL) or combination (LBP = 2 mg/mL, LJP = 1 mg/mL and RSV = 100 μM) were assessed.

**Results:**

RSV, LBP, LJP, or a combination of RSV, LBP, and LJP added to the freezing medium significantly improved sperm progressive motility, plasma membrane integrity, acrosome integrity, and mitochondrial activity during the cryopreservation process. Furthermore, the activities of glutathione peroxidase and superoxide dismutase were also improved. The levels of reactive oxygen species and malondialdehyde in semen were notably reduced. Hyaluronidase activity and acrosin activity were significantly increased in LBP-treated sperm. However, sperm total motility and DNA integrity were not significantly different between the groups.

**Conclusions:**

RSV (50 μM) or LBP (2 mg/mL) are the best candidate antioxidants for inclusion in the freezing medium to improve the quality of giant panda spermatozoa during semen cryopreservation.

## Background

Giant pandas (*Ailuropoda melanoleuca*) are one of the vulnerable animals in the world, and have survived on earth for at least eight million years [1, 2]. The Qinling giant panda (*Ailuropoda melanoleuca qinlingensis*), a subspecies of the giant panda, is a precious species because of its unique morphological characteristics and genetic features. From the fourth investigation report in 2015 by China’s State Forestry Administration, approximately 345 individuals inhabited the Qinling Mountains, accounting for 18.51% of all wild giant pandas in China. Low mating opportunity is the main factor driving hypofertility in giant pandas, especially in captive-bred giant pandas, which have fewer chances of finding a suitable mate compared with wild giant pandas. Therefore, only 10 % of the captive-bred giant pandas can mate naturally. Artificial insemination has become an effective biotechnology approach in conservation efforts of the giant panda; however, the efficiency of semen freezing is one of the major problems affecting the success of artificial insemination [3, 4]. Ultra-low temperature cryopreservation may inflict damage to sperm cells; therefore, there is an increasing demand for improving semen cryopreservation methods for giant panda semen. This will be beneficial in maintaining the number of Qinling giant pandas, establishing a germplasm repository for the Qinling subspecies, and improving their conservation status.

Increasing evidence has shown that sperm cryopreservation induces the overproduction of reactive oxygen species (ROS), which are major deleterious factors affecting sperm quality [5]. Excessive ROS production leads to drastic changes in sperm membranes because natural antioxidants in the seminal plasma are diluted in the freezing medium before the cryopreservation process [6]. Recently, the addition of antioxidants to the sperm freezing medium for humans, horses, beers, dogs, goats, cattle, and boars has been demonstrated to significantly enhance sperm viability [7–11]. Multiple studies have confirmed that resveratrol (RSV), lycium barbarum polysaccharide (LBP), and laminaria japonica polysaccharides (LJP) can reduce excessive ROS production, thus limiting cryodamage [12–14]. RSV is a polyphenol found in plants such as grapes [15]. Recent studies have shown that RSV modulates lipid metabolism and has anti-inflammatory, antioxidant, anti-allergic, and anti-cancer properties. Additionally, RSV displays protective effects against free radicals, cardiovascular diseases, and allergies [16]. Evidence from animal model studies on male fertilization have confirmed that RSV plays a promoting role in spermatogenesis by stimulating the hypothalamic-pituitary gonadal axis [17]. In addition, RSV suppresses germ cell apoptosis, enhances penile erection, elevates steroidogenesis, and facilitates sperm motility and spermatogenesis in rats and mice [18]. LBP extracted from barbarums has been shown to improve the sperm quality. Moreover, the addition of LBP to the freezing medium not only increases the activity of antioxidant enzymes to inhibit apoptosis, but also enhances spermatogenesis in male mice with type 1 diabetes [19]. Multiple studies have indicated that LJP, a polysaccharide extracted from seaweed, has anti-coagulant, anti-arteriosclerosis, antitumor, and antiviral properties. In addition, studies have demonstrated that LJP can be added to the freezing medium, acting as a ROS scavenger to improve sperm quality during the freeze-thawing process [20].

In this study, we evaluated the potential effects of cryopreservation with RSV, LBP, or LJP on sperm motility, sperm plasma membrane integrity, acrosome integrity, mitochondrial activity, DNA integrity, antioxidant activities, and fertilization capacity. A further goal of this study was to provide effective antioxidants added in freezing medium to improve sperm resistance to cryopreservation.

## Results

### Antioxidant supplementation improves sperm motility of Qinling giant panda during cryopreservation

**Fig. 1:**
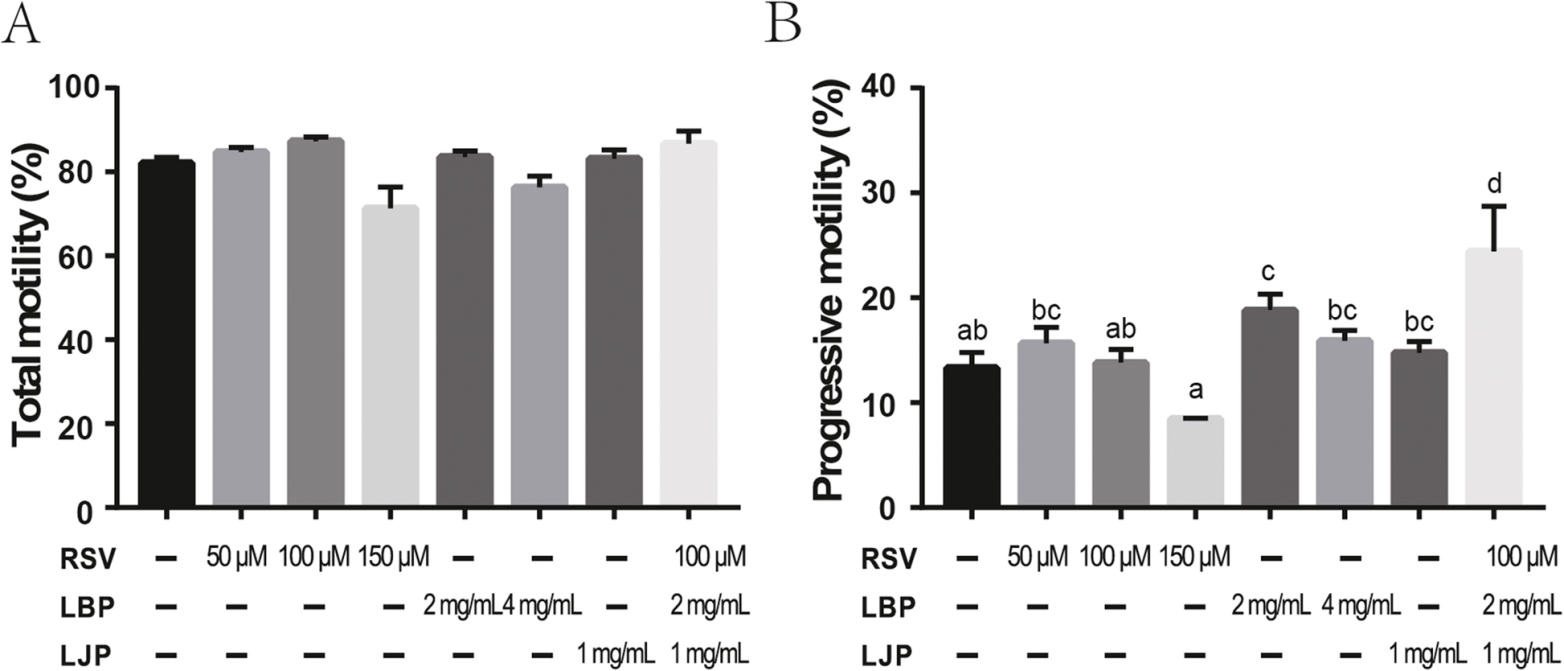
Total and progressive motility of giant panda spermatozoa treated with antioxidants. (**A**) The total motility of freeze-thawed sperm in the presence of antioxidants. (**B**) Sperm progressive motility in freezing medium supplemented with antioxidants. Values are presented as mean ± SEM (n = 3). Letters a, b, c, and d indicate significant difference levels, *P*<0.05

To explore the roles of antioxidants (RSV, LJP, and LBP) in giant panda sperm resistance to cryopreservation, we first assessed sperm total and progressive motility. We found no enhanced effect on the total motility of sperm exposed to RSV, LBP, or LJP alone at the indicated concentrations. Furthermore, the combination of RSV (100 μM), LBP (2 mg/mL), and LJP (1 mg/mL) also did not change the total motility of freeze-thawing sperm when compared with the control (Fig. 1A, *P* > 0.05). As shown in Fig. 1B, RSV alone had no effect on sperm progressive motility. Similarly, there was no significant difference between the LJP and control group, or the LBP (4 mg/mL) group (*P* > 0.05). However, addition of LBP (2 mg/mL) in freezing medium improved progressive motility compared with the control (*P* < 0.05). Furthermore, the combination of RSV (100 μM), LBP (2 mg/mL), and LJP (1 mg/mL) notably improved sperm progressive motility after freeze-thawing process compared with the control (*P* < 0.05). Notably, a better post-thaw sperm progressive motility was observed in the combination group than in the LBP group (2 mg/mL) (*P* < 0.05). These data indicated that supplementation with antioxidants did not change sperm total motility, but did improve sperm progressive motility.

### Antioxidant supplementation improves plasma membrane and acrosome integrity of Qinling giant panda sperm during cryopreservation 

**Fig. 2:**
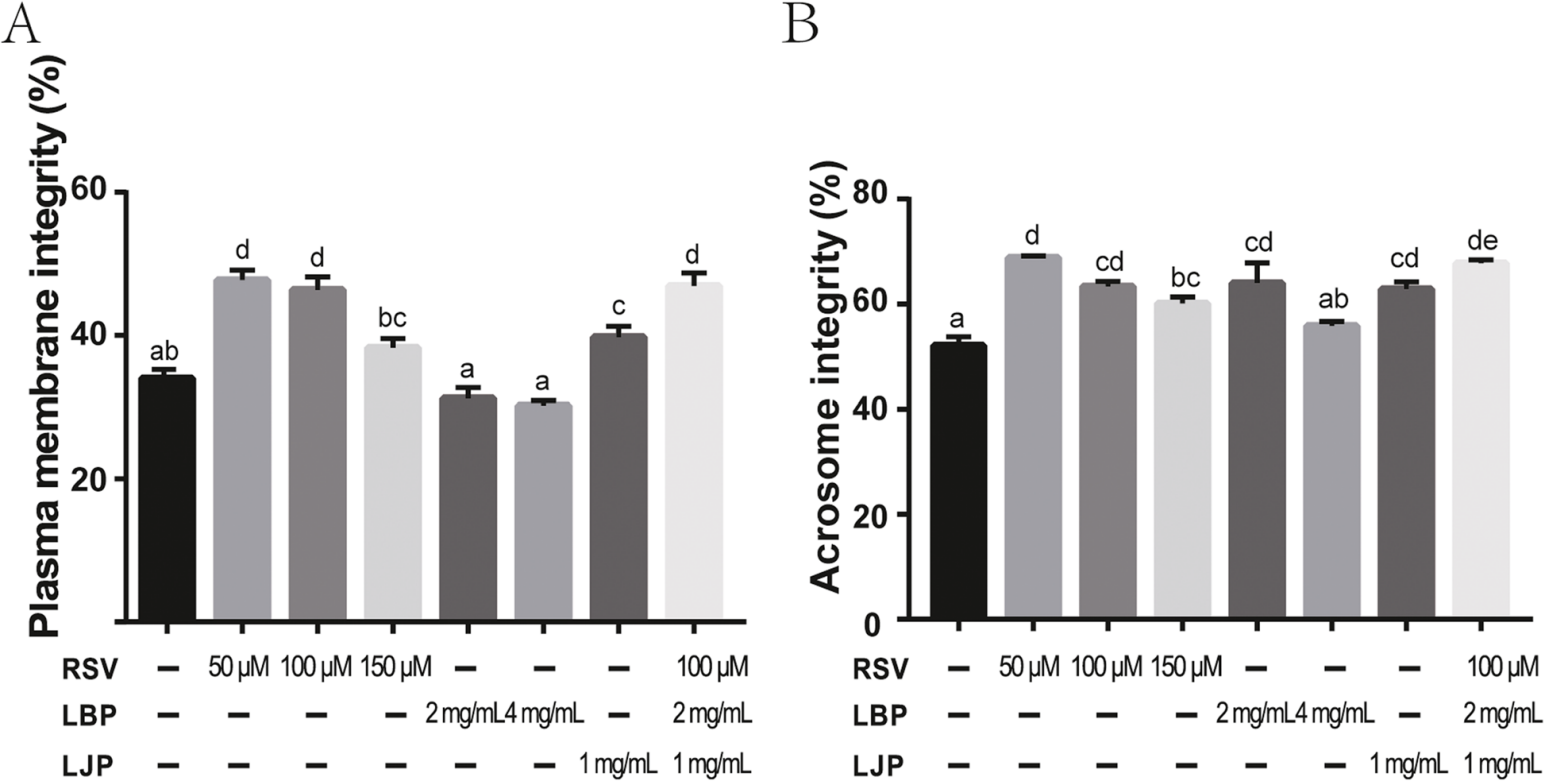
The effects of antioxidants on plasma membrane integrity and acrosome integrity of freeze-thawed spermatozoa. (**A**) Plasma membrane integrity of spermatozoa assessed by HOST staining. (**B**) The acrosome integrity in post-thaw spermatozoa assessed by FITC-PNA staining. Data are shown as mean ± SEM; Letters a, b, c, d and e indicate significant difference levels, *P*<0.05.

Increasing evidence has shown that plasma membrane and acrosome integrity are associated with sperm motility [6]. Thus, we detected plasma membrane integrity and acrosome integrity in sperm treated with antioxidants. We found that supplementation with 50 μM RSV and 100 μM RSV markedly improved the plasma membrane integrity (Fig 2A, *P* < 0.05), while there was no significant difference between the 50 μM RSV and 100 μM RSV treatment groups. Correspondingly, LJP (1 mg/mL) showed significant protective effects (Fig. 2A, *P <* 0.05). In addition, the combination of RSV (100 μM), LBP (2 mg/mL), and LJP (1 mg/mL) protected the plasma membrane integrity compared with the control (Fig. 2A, *P* < 0.05), yet showed no difference in the protection of plasma membrane integrity in comparison with RSV (50 / 100 μM) alone (Fig. 2A, *P* > 0.05). However, LBP had no significant protective effect at all (Fig. 2A, *P* > 0.05). These results suggested that RSV alone, LJP alone, and the combination had the ability to protect the plasma membrane integrity during cryopreservation, and 50 μM of RSV was the optimal dose. As shown in Fig. 2B, we found that freezing medium with RSV alone improved acrosome integrity (*P* < 0.05). Furthermore, 1.0 mg/mL LJP alone and 2.0 mg/mL LBP alone improved acrosome integrity (*P* < 0.05). As expected, the combination of RSV (100 μM), LBP (2 mg/mL), and LJP (1 mg/mL) increased acrosome integrity compared with the control (*P* < 0.05). Notably, RSV (50 μM) and the combination were more effective than RSV (100 μM), RSV (150 μM), LBP (2 mg/mL), and LJP (1 mg/mL) alone. These results suggested that RSV, LJP, LBP, and their combinations at certain concentrations could protect acrosome integrity during cryopreservation.

### Antioxidant supplementation improves mitochondrial activity of Qinling giant panda sperm during cryopreservation

**Fig. 3:**
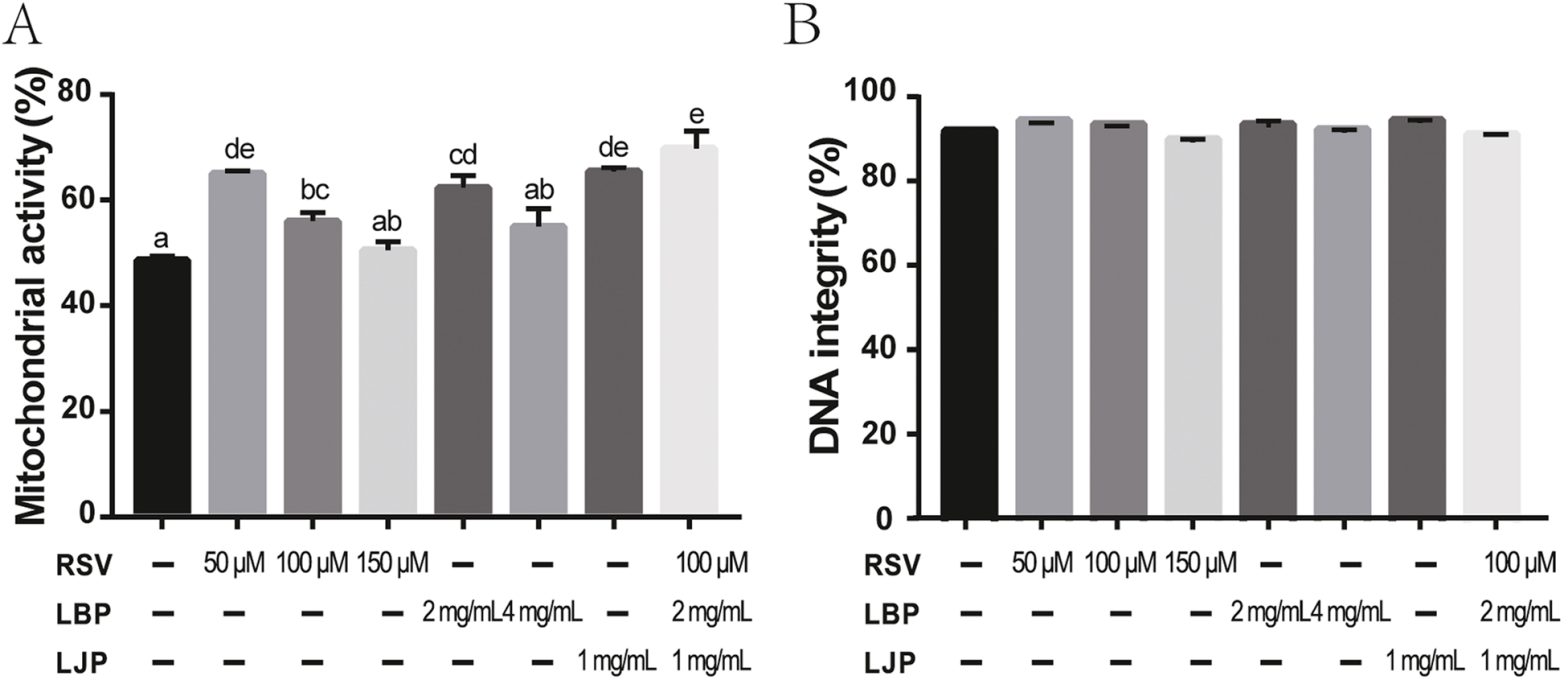
Evaluation of the effect of antioxidants on mitochondrial activity and DNA integrity in giant panda spermatozoa. (**A**) Sperm mitochondrial activity assessed by staining with PI and Rh123. (**B**) DNA integrity evaluated using AO staining. Values are shown as mean ± SEM (n = 3). Letters a, b, c, d and e indicate significant difference levels, *P*<0.05.

To analyze the further protective effect of antioxidants, mitochondrial activity and DNA integrity were evaluated using PI/Rh123 staining and AO staining, respectively. The results showed that 50 μM RSV and 100 μM RSV significantly protected mitochondria from freeze-thawing injury (Fig. 3A, *P* < 0.05), and mitochondrial activity was significantly increased in the freezing medium with addition of 2 mg/mL LBP or 1 mg/mL LJP alone (Fig. 3A, *P* < 0.05). Furthermore, the combination of RSV (100 μM) and LBP (2 mg/mL) with LJP (1 mg/mL) significantly improved the mitochondrial activity of sperm when compared with the control (Fig. 3A, *P* < 0.05). However, no significant effect on mitochondrial activity was observed in RSV (150 μM) or LBP (4 mg/mL) groups. From the above results, 50 μM RSV, 1.0 mg/mL LJP, and the combined addition showed best protective effect in terms of mitochondrial activity (Fig. 3A, *P* < 0.05). As shown in Fig. 3B, no significant effect on the DNA integrity of sperm was observed after treatment with RSV, LJP, LBP, or the combined addition compared with the control (*P* > 0.05). Collectively, these data suggested that 50 μM RSV and LJP (1 mg/mL) were the best candidates to improve the mitochondrial activity of sperm in freezing media.

### Antioxidant supplementation mitigates ROS levels and improves MDA, SOD, and GPX activity in cryopreserved sperm of Qinling giant panda

**Fig. 4:**
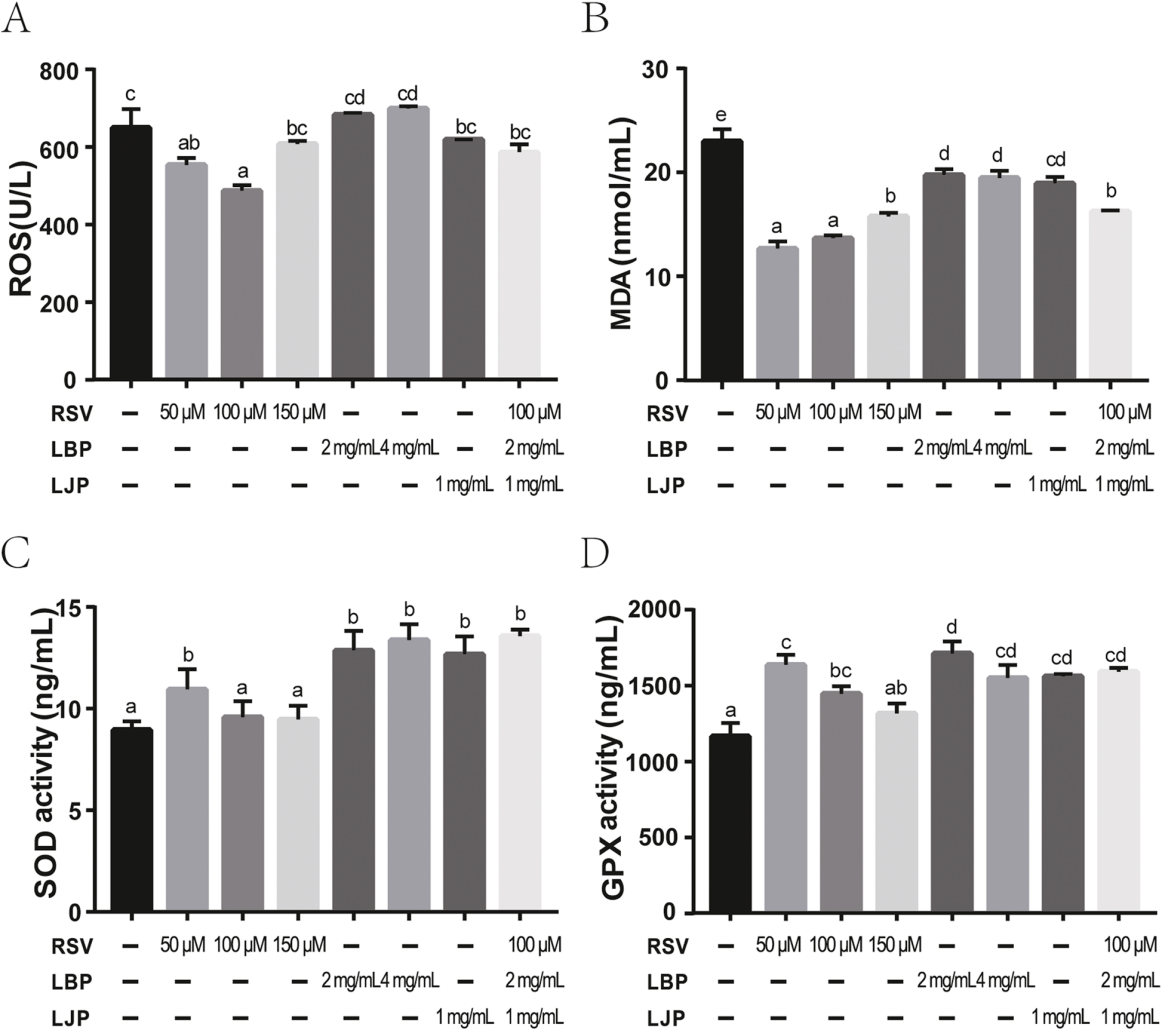
Antioxidants enhanced oxidation resistance of giant panda spermatozoa during the freeze-thawing process. The levels of ROS (**A**), MDA (**B**), SOD (**C)** and GPX (**D**) in giant panda semen treated with antioxidants or control were determined by ELISA. The values are shown as means ± SEM of three independent repeats. Letters a, b, c and d indicate significant difference levels, *P*<0.05

To detect the protective capacity of antioxidants in thawed semen, antioxidant indices were determined using ELISA kits. Reactive oxygen species (ROS) levels were found to be significantly decreased in freezing media supplemented with 50 μM RSV and 100 μM RSV (Fig. 4A, *P* < 0.05), but no significant change was found in the level of ROS after treatment with 150 μM RSV, LBP, LJP, or their combination. In addition, resistance to ROS was observed in the 50 μM and 100 μM RSV groups, but better in the 100 μM RSV group. As shown in Fig. 4B, all freezing media with RSV, LBP, LJP, or the combined addition had a significant protective effect against high levels of malondialdehyde (MDA) during freeze-thawing (*P* < 0.05). The effective protection of RSV (50 μM) and RSV (100 μM) was the highest (Fig. 4B, *P* < 0.05). Addition of 50 μM RSV, LBP alone, LJP alone and the combined addition contributed to increased superoxide dismutase (SOD) levels (Fig. 4C, *P* < 0.05). However, RSV (100 μM and 150 μM) did not have effect on SOD levels compared with the control (Fig. 4C, *P* > 0.05). Analysis of glutathione peroxidase (GPX) levels revealed that the facilitating effect of 50 μM RSV alone and 100 μM RSV alone was observed in sperm cryopreservation (Fig. 4D, *P* < 0.05), but RSV (150 μM) did not enhance GPX levels. LBP and LJP alone also improved the level of GPX in comparison with the control (Fig. 4D, *P* < 0.05). Similarly, the combination of RSV (100 μM), LBP (2 mg/mL), and LJP (1 mg/mL) significantly increased the level of GPX compared to the control (Fig. 4D, *P* < 0.05). Among the treatments, LBP (2 mg/mL) was the best candidate in elevating GPX levels. Collectively, these data indicated that antioxidants in the freezing medium decreased ROS and MDA levels while increased SOD and GPX levels.

### Supplementation of freezing media with antioxidants improves fertilization capacity of sperm of Qinling giant panda

**Fig. 5:**
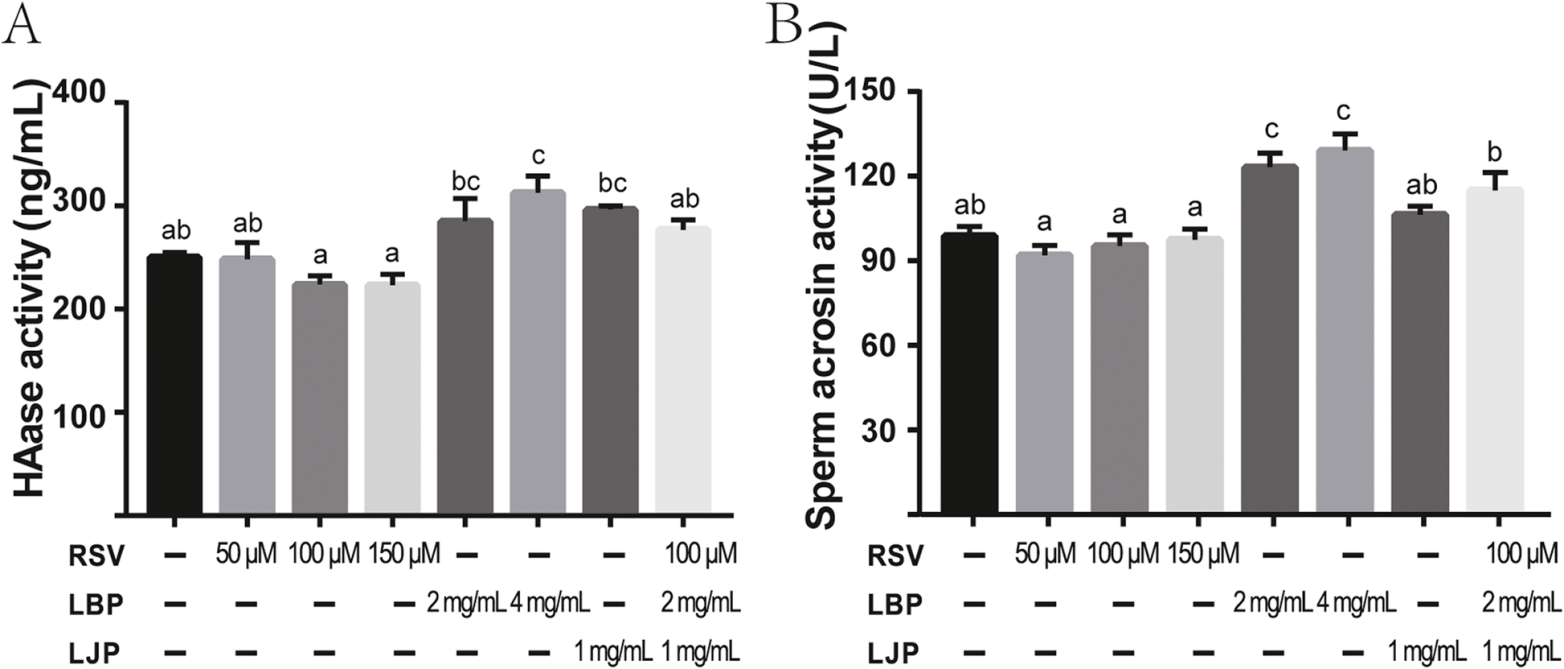
Improvements in fertilizing capability in giant panda spermatozoa. (**A**) Sperm HAase content determined by ELISA. (**B**) Acrosin activity of spermatozoa measured by ELISA. All the experimental values are shown as the means ± SEM of three repeats. Letters a, b and c indicate significant difference levels, *P*<0.05.

To analyze the fertilization ability of giant panda sperm after cryopreservation, protease assays were performed using ELISA. As shown in Fig. 5A, a significant difference in the hyaluronidase (HAase) content in sperm was found after treatment with LBP (4 mg/mL) compared with the control (Fig. 5A, *P* < 0.05). However, no enhancement was observed in sperm treated with RSV, LBP (2 mg/mL), LJP, or their combination (Fig. 5A, *P* > 0.05). As shown in Fig. 5B, supplementation of LBP with doses of 2 mg/mL and 4 mg/mL exerted a dramatic cryoprotective effect on sperm acrosomal protease (ACE) activity (*P* < 0.05), and the difference in sperm ACE activity between 2 mg/mL and 4 mg/mL was not significant (Fig. 5B, *P* > 0.05). However, RSV, LJP, and the combined addition did not promote sperm ACE activity. Overall, these data indicated that the addition of LBP to the freezing medium improved the fertilization capacity.

## Discussion

Giant pandas are one of the vulnerable species endemic to China, and adaptive for protection of the Qinling subspecies is the most pressing challenge. Apart from the establishment of nature reserves, semen cryopreservation of giant pandas is an effective approach to facilitate reproduction and sustain biodiversity. However, the negative effect of cryopreservation on the quality of giant panda sperm is inevitable. Substantial evidence has suggested that cryopreservation severely compromises plasma membrane fluidity, antioxidant ability, acrosome enzyme activity, mitochondrial potential, and motility, thereby affecting the sperm quality. Many researchers have investigated methods to protect the sperm from cryo-induced damage. Studies have further demonstrated that the addition of cryoprotective agents is important in reducing damage to sperm during cryopreservation. In cryoprotectant type, silicane-coated silica colloid particles were added to the freezing medium to improve the ability of spermatozoa fertilization of giant panda [21]. INRA96 freezing dilutions increased the quality of giant panda semen after thawing, compared to the TEST extender [22]. In addition, cryoprotectants, such as glycerol, sucrose, vitamin B12, vitamin E, vitamin C, dimethyl sulfoxide (DMSO), and trehalose were added to the freezing medium. At the transcript level, differentially expressed lncRNAs, miRNAs and mRNAs were analyzed in frozen and fresh sperm of giant pandas using high-throughput sequencing technology [23, 24]. The above evidence showed that the sperm of giant pandas suffered cryo-injury [25]. Previous reports have shown that RSV, LBP, and LJP added to semen improved the quality of sperm [7]. While the protective effect of antioxidants on ruminant sperm has been widely investigated, few studies have explored the optimum doses of LBP, LJP, and RSV for giant panda sperm cryopreservation. To develop new cryopreservation techniques and methods, novel findings regarding freezing medium of giant panda supplemented with antioxidants are in high demand. Because the cytotoxic effects rise with high concentrations, the optimal dose and concentration of LBP, LJP, and RSV were referenced from the dose in cattle, sheep, pigs, and other animals. In our study, seven groups of antioxidants were added to the freezing medium and positive effect on sperm quality was observed. This study provides an important reference for future improvements in cryopreservation of giant panda sperm.

Pioneering work previously showed that supplementation of buffalo semen freezing medium with RSV (50 μM) could markedly improve the post-thaw quality of sperm [26]. Moreover, the addition of RSV (10 μM and 50 μM) to the freezing medium prior to cryopreservation also attenuated cryodamage and enhanced the motility of goat sperm [7]. Research has shown that 2 mg/mL LBP + 1 mg/mL LJP could significantly improve the quality of Cashmere goat sperm [27]. In our study, we detected the protective effects of LBP, LJP, and RSV on giant panda sperm after cryopreservation. Similarly, we confirmed that supplementation of the freezing medium with 2 mg/mL LBP or a combination was effective at protecting giant panda sperm from cryoinjury. However, RSV and LJP alone had no protective effect on giant panda sperm motility. Interestingly, the dose of LBP applied to Cashmere goat spermatozoa can be considered to improve giant panda sperm motility. During the freeze-thaw process, the plasma membrane fluidity and permeability increased. The redistribution of antioxidant enzymes, phospholipids, and cholesterol is observed in the sperm plasma membrane and is responsible for structural and functional changes. In addition, plasma membrane integrity, which is involved in metabolism and osmotic pressure, is a critical factor in fertility [28]. Recently, evidence has confirmed that cryopreservation are detrimental to plasma membrane integrity and acrosome integrity in bulls, boars, rams, and sheep [26, 29–31]. To improve plasma membrane integrity and acrosome integrity, 50 μM RSV was found to be an effective antioxidant for boar semen cryopreservation [13]. Supplementation of tris citric acid extender with 100 μM RSV significantly enhanced the plasma membrane integrity of buffalo [32]. Furthermore, LJP and LBP exert protective effects on plasma membrane integrity and acrosome integrity [27]. Based on the antioxidant function of antioxidants, we further investigated whether antioxidants exerted a cryoprotective effect on giant panda sperm. Our study showed that 50 μM RSV, 100 μM RSV, LJP alone and their combination had a cryoprotective effect on giant panda sperm plasma membrane integrity and acrosome integrity, which was consistent with the above-mentioned report. In addition, 50 μM RSV and LBP (2 mg/mL) were beneficial to acrosome integrity. Recently, evidence from cryopreservation studies has confirmed that sperm with damaged DNA have been detected in cats, humans, and bovines [33–35]. Furthermore, antioxidants, extenders, and new approaches for humans have been used to improve sperm chromatin quality during freezing [33–35]. However, there were no protective effects of RSV, LBP, and LJP on the DNA integrity of giant panda semen after cryopreservation in our study.

Mitochondrial ATP for energy homeostasis is generated by oxidative phosphorylation and ATP synthases, which plays a vital role in sperm motility [36]. Mitochondria are the primary organelles responsible for ROS production due to disturbances in the electron transport chain. In addition, the capacity of antioxidant enzymes against oxidative stress is significantly reduce because their abundance is diluted and thus have a negative effect on the cryopreservation results [37]. Therefore, excessive ROS levels can impair mitochondrial proteins, resulting in severely damaged mitochondrial activity [6, 38]. Recently, evidence has shown that the addition of RSV, LBP, and LJP to freeze-thawed semen could significantly improve the mitochondrial activity of sperm [7, 13, 27, 38]. In our study, we verified that the addition of 50 μM RSV, 100 μM RSV, 2 mg/mL LBP, 1 mg/mL LJP or the combined addition into the freezing medium improved mitochondrial activity of sperm, which was consistent with a previous study [13]. Previous reports have shown that cryopreservation is harmful to mitochondrial function and increases the production of ROS, and excessive production of ROS attacks polyunsaturated phospholipids and trigger elevation of MDA levels. In addition, the antioxidant activity of SOD and the level of GPX are reduced during cryopreservation. Finally, the semipermeable properties of the membrane are affected by peroxidative attack. However, the addition of RSV, LJP, and LBP to semen reduces the negative effects of excessive ROS levels [27, 38–40]. Consistent with a previous study, we further verified that 50 μM RSV and 100 μM RSV inhibited the production of ROS. In addition, our results showed that the addition of antioxidants prior to freezing protected sperm from cryodamage induced by MDA, whereas 50 μM RSV was the best potential candidate as a cryoprotective agent. Recently, accumulating evidence has revealed that RSV, LJP and LBP could be beneficial by modulating activity of SOD and GPX [27, 41]. Our study verified that supplementation of the freezing medium with 50 μM RSV , 2 mg/mL LBP, 1 mg/mL LJP, and combined addition significantly enhanced activity of SOD and GPX for oxygen radical (O_2_^−^) scavenging. Therefore, supplementation of the freezing medium with antioxidants improved the enzymatic antioxidant activities of giant panda sperm.

In mammals, spermatozoa penetrate the zonal pellucida through the action of acrosomal enzymes. Among acrosomal enzymes, acrosin is identified as the most important factor, with a strong hydrolyzing activity. Evidence has shown that sperm mitochondrial activity affects human sperm acrosin activity [42]. In addition, HAase is important for digesting hyaluronic acid during the acrosome reaction process [43]. Therefore, HAase activity in sperm is responsible for fertilization. In our study, sperm ACE and HAase levels were improved in the 4 mg/mL LBP treated group, and 2 mg/ml LBP had protective effects on sperm ACE.

## Conclusion

In summary, our study revealed that freezing medium supplemented with LJP, LBP, RSV, and their combination enhanced sperm motility, sperm plasma membrane integrity, acrosome integrity, and mitochondrial activity by increasing antioxidant enzyme levels and suppressing ROS production during the freezing process. 50 μM RSV and 2.0 mg/mL LBP could be potential candidates as supplementation of the freezing medium against cryodamage to giant panda sperm. The study provides a reference for the improvement of Qinling giant panda semen cryopreservation.

## Material and Methods

### Semen collection

Semen from four sexually mature male giant pandas (aged 12–17 years) housed in Shaanxi Wild Animal Research Center (SWARC; Louguantai, Zhouzhi County, Xi’an city, 34°06 N, 108°32 E) were collected via electrostimulation during the breeding season (from March to May in spring). The giant pandas were anesthetized by injection with ketamine hydrochloride (8 mg/kg, IM) and halothane after 12 h of fasting, and the manually protruding penis was cleaned with pre-warmed sterile saline. The feces was then cleaned from the rectum using enema. To collect semen, the giant pandas were then subjected to 2-3 times electroejaculations (10–90 mA, 5 s) with an interval of 2 s by a probe treated with carboxymethyl cellulose gel. Approximately 5 mL of fresh semen was collected into 37 °C pre-warmed sterile cups from each one. This process was performed in compliance with the Wildlife Protection Law of the People’s Republic of China and Regulations of Shaanxi Province on the Protection of Wild Animals and Plants. All protocols were approved by the Committee for the Ethics on Animal Care and Experiments in Northwest A&F University (2017ZX08008005).

### Experimental design

Fresh semen samples were diluted with an extender (Irvine Scientific 90129) at a 1:1 (v:v) ratio in 15 mL tubes placed in a beaker of water at 37 °C. Subsequently, the tubes were incubated at 4 °C for 3 h. After that, the original cryoprotectant agent (Irvine Scientific 90128) was used to dilute the semen to a final concentration of 400 ×10^6^ spermatozoa/mL, with a final glycerin concentration of 3.33%. Diluted semen was equilibrated at 4 °C for 1 h and aliquoted into eight sets of pre-cooled tubes. Prior to cryopreservation, three sets were supplemented with 50 μM RSV, 100 μM RSV, or 150 μM RSV. Three sets were supplemented with LBP (2 and 4 mg/mL) and LJP (1 mg/mL), respectively. and another set was supplemented with a combination of antioxidants, including LJP (1 mg/mL), RSV (100 μM), and LBP (2 mg/mL). In addition, a set without any antioxidants was set as the control. Finally, diluted semen aliquots were packed into pre-labeled and pre-cooled 250 μL straws, frozen in liquid nitrogen vapor for 2 min (at 7.5 cm above liquid nitrogen for 1 min, 2.5 cm above liquid nitrogen for 1 min), and stored in nitrogen for long-term preservation [25]. For thawing, frozen straws were removed from liquid nitrogen and immediately placed in a water bath at 37 °C. After shaking for 60 s, the thawed semen was transferred into a 1.5 mL tube for subsequent experiments.

### Semen evaluation

#### Sperm motility test

After thawing, semen of giant panda was incubated at 37 °C, immediately. Prior to evaluation, the thawed semen was placed in the pre-warmed makler counting chamber (Sefi-Medical, Haifa, Israel) at 37 °C. Subsequently, the total and progressive motility of spermatozoa were examined using an automatic analyzer (Naturegene Sperm Tracker, ANIMAL HST, NatureGene Corp. USA) following the manufacturer’s recommendations. Finally, the total and progressive motility of 200 spermatozoa from 5 randomized microscopic fields were evaluated.

#### Sperm plasma membrane integrity test

Following the findings of prior studies, the integrity of the sperm plasma membrane was assessed using HOST test [44]. Briefly, hypotonic solutions (25 mM sodium citrate, 75 mM fructose, sterile water) were mixed with rapidly thawed semen of giant pandas, and the mixture was incubated at 37 °C for 30 min. A total of 200 spermatozoa were counted under an inverted microscope (Soi, Nikon, JPN) to identify the percentage of sperm with swelling tails.

### Acrosome integrity assessment

Acrosome integrity was assessed by fluorescein isothiocyanate-labeled peanut agglutinin (FITC-PNA) staining (Vector Laboratories, USA) [45]. Thawed semen was prepared for sperm smears to a final concentration of 1 × 10^5^/mL and kept dry. The cells were then fixed with methanol for 10 min and incubated with 30 μL FITC-PNA (100 μg/mL) at 37 °C for 30 min in an environment of light avoidance. Sperm smears were then sealed with a colorless nail polish after washed with PBS in triplicate. Finally, more than 200 sperm cells were evaluated using fluorescence microscopy (Axio Observer 3, ZEISS, GER).

### Mitochondrial activity analysis

Tubes containing 1 μL PI (0.02 mg/mL in PBS solution) (Solarbio, Beijing, China) and 1 μL Rhodamine Rh123 (0.2 mg/mL in DMSO) (Solarbio, Beijing, China) were stored in dark at room temperature for 10 min and carefully supplemented with 50 μL suspension of sperm that was freeze-thawed for 30 min [46]. Sperm mitochondrial activity was estimated using an inverted microscope (Soi, Nikon, JPN) at 400× magnification after sperm smears were prepared with 10 μL of the incubated sample. PI negative and Rh123 positive sperm were identified as live sperm with high mitochondrial membrane potential (ΔΨm).

### Evaluation of DNA integrity

DNA integrity was assessed by acridine orange (AO) staining (Solarbio, Beijing, China) [47]. The sperm were washed three times with PBS and then suspended in PBS at a concentration of 2 × 10^4^/mL. The sperm suspension was placed on clean glass slides and air-dried for the preparation of smears. The smears were then rinsed in a fixative solution (3:1, absolute ethanol: glacial acetic acid) for 5 min. After fixation, the slides were stained with AO solution for 10 min and thoroughly washed with distilled water. Finally, the air-dried smears were sealed with paraffin oil and analyzed using fluorescence microscopy (Axio Observer 3, ZEISS, GER). At least 300 sperm were counted per smear.

### ELISA estimation of ROS, MDA, SOD, GPX, HAase and ACE levels

The reagent kits were equilibrated at room temperature for 30 min. ROS, MDA, SOD, GPX, HAase and ACE levels were determined by ELISA (Jining, Shanghai, China) in triplicate following the manufacturer’s instructions. The optical density of each sample was measured using a multifunctional microplate reader (SPARK, TECAN, CH) at a wavelength of 450 nm. Subsequently, the levels of ROS, MDA, SOD, GSH, HAase and ACE were calculated against each standard curve.

### Statistical analysis

All experiments were replicated at least three times for each group. Data were expressed as means ± SEM. The data were analyzed with one-way ANOVA followed by LSD using the SPSS Software and the homogeneity of variance was analyzed by Bartlett's test. Statistical significance was set at *P* < 0.05.

## Data Availability

The datasets supporting the conclusions of this study are available from the corresponding author.

## Abbreviations

LBP: Lycium barbarum polysaccharide
LJP: Laminaria japonica polysaccharides
RSV: Resveratrol
ROS: Reactive oxygen species
SOD: Superoxide dismutase
GSH-PX: Glutathione
MDA: Malondialdehyde
HAase: Hyaluronidase
ACE: Acrosomal protease
